# Identification of Strain-Specific Sequences That Distinguish a Mycoplasma gallisepticum Vaccine Strain from Field Isolates

**DOI:** 10.1128/JCM.00833-16

**Published:** 2016-12-28

**Authors:** Camir Ricketts, Larissa Pickler, John Maurer, Saravanaraj Ayyampalayam, Maricarmen García, Naola M. Ferguson-Noel

**Affiliations:** a Poultry Diagnostic and Research Center, University of Georgia, Athens, Georgia, USA; b Institute of Bioinformatics, University of Georgia, Athens, Georgia, USA; c Department of Veterinary Medicine, Universidade Federal do Parana, Curitiba, PR, Brazil; d CAPES Foundation, Ministry of Education of Brazil, Brasília, DF, Brazil; University of Tennessee

**Keywords:** strain typing, ts-11, ts-11-like, PCR, genomes, Mycoplasma gallisepticum

## Abstract

Despite attempts to control avian mycoplasmosis through management, vaccination, and surveillance, Mycoplasma gallisepticum continues to cause significant morbidity, mortality, and economic losses in poultry production. Live attenuated vaccines are commonly used in the poultry industry to control avian mycoplasmosis; unfortunately, some vaccines may revert to virulence and vaccine strains are generally difficult to distinguish from natural field isolates. In order to identify genome differences among vaccine revertants, vaccine strains, and field isolates, whole-genome sequencing of the M. gallisepticum vaccine strain ts-11 and several “ts-11-like” strains isolated from commercial flocks was performed using Illumina and 454 pyrosequencing and the sequenced genomes compared to the M. gallisepticum R_low_ reference genome. The collective contigs for each strain were annotated using the fully annotated Mycoplasma reference genome. The analysis revealed genetic differences among *vlhA* alleles, as well as among genes annotated as coding for a cell wall surface anchor protein (*mg0377*) and a hypothetical protein gene, *mg0359*, unique to M. gallisepticum ts-11 vaccine strain. PCR protocols were designed to target 5 sequences unique to the M. gallisepticum ts-11 strain: *vlhA*3.04a, *vlhA*3.04b, *vlhA*3.05, *mg0377*, and *mg0359*. All ts-11 isolates were positive for the five gene alleles tested by PCR; however, 5 to 36% of field isolates were also positive for at least one of the alleles tested. A combination of PCR tests for *vlhA*3.04a, *vlhA*3.05, and *mg0359* was able to distinguish the M. gallisepticum ts-11 vaccine strain from field isolates. This method will further supplement current approaches to quickly distinguish M. gallisepticum vaccine strains from field isolates.

## INTRODUCTION

Mycoplasmas are bacteria characteristic for their lack of a cell wall, small size, and small genome (1). These pathogens are evolutionarily related to the low-%GC Gram-positive Clostridiales (2), and they are highly host specific and tend to inhabit mucosal surfaces within their host (1). Within the poultry industry, the ability of Mycoplasma gallisepticum to infect the respiratory and reproductive tracts of several avian species has made it a pathogen of great economic concern (3). In an effort to manage the disease, M. gallisepticum strain F (4, 5) ts-11 (6, 7), and 6/85 (8) live vaccines were developed. The F strain is a naturally attenuated field isolate that was first discovered in the 1950s, while ts-11 and 6/85 were commercially produced using serial passage or chemical mutagenesis (7). These vaccine strains exhibit various degrees of effectiveness and safety (9–11), and there is evidence that some of these M. gallisepticum vaccine strains can revert to virulence (9, 12, 13). Currently, the genetic basis behind the attenuation of the vaccine strains is not well understood. In addition, strain differentiation among vaccine strains and natural isolates has proven complex (14–17), making it difficult to determine if avian mycoplasmosis infection in vaccinated flocks is due to infection with an M. gallisepticum strain type similar to the vaccine (18), reversion of the vaccine strain (9), or mixed infection with the vaccine and a related strain type (19).

M. gallisepticum isolates vary widely in their relative degrees of pathogenicity in animal challenge experiments, depending on the route of infection and the number of *in vitro* passages (20–22). Serial passaging of this organism *in vitro* has been used to create attenuated strains for use as vaccines (7), but the likelihood of reversion to wild-type virulence is inherent to this attenuation method. It has also been difficult to differentiate vaccine M. gallisepticum strains from some field isolates (12, 13, 23). This is important in order to differentiate M. gallisepticum field infections from vaccine exposures in a timely manner and, also, in order to assess the reversion to virulence of vaccine strains.

The genome of Mycoplasma is relatively small compared to other bacterial genomes; the average size of Mycoplasma genome is 1.0 Mb (the range is from 580 kb to 1,380 kb) (24), one quarter of the average size of an Escherichia coli genome. Historically, Mycoplasma genitalium was the second complete bacterial genome ever published (25), and since then, over 50 Mycoplasma genomes, including pathogens of humans, animals, and plants, have been reported, including M. gallisepticum (26). Despite these facts, compared to other pathogens, few virulence-related genes have been identified in *M. gallisepticum*. GapA (a primary cytadhesin) and CrmA (an accessory cytadhesin) mediate the attachment of this pathogen to the respiratory epithelium of the host (27). VlhA is a surface lipoprotein that undergoes phase variation; changing the bacterial surface architecture and allowing the mycoplasmas to escape immune surveillance (28, 29). There are also several metabolic pathways important to M. gallisepticum virulence; M. gallisepticum has been shown to depend on the dihydrolipoamide dehydrogenase (Lpd), a component of the pyruvate dehydrogenase complex, for host colonization and pathogenesis (30). The expression of MalF, an ABC transporter, has also been shown to be essential for persistence (31).

In 2007, several broiler breeder flocks in northeastern Georgia were vaccinated with ts-11 vaccine to control an ongoing M. gallisepticum outbreak. Between 2008 and 2011, severe respiratory disease associated with M. gallisepticum infection was observed in the broiler progeny of several ts-11-vaccinated breeder flocks. M. gallisepticum isolates from the broilers and their parents were indistinguishable from the ts-11 vaccine strain by the genotyping methods used and were termed “ts-11-like” isolates (9). The epidemiology of the outbreaks, as well as genotyping and pathogenicity results, indicate that an increase in virulence and vertical transmission of ts-11 vaccine occurred and that the ts-11-like isolates were very likely revertants derived from ts-11 vaccine (9, 16, 32).

In order to identify M. gallisepticum ts-11-specific marker alleles, whole-genome sequencing was used. M. gallisepticum DNA was sequenced using both Illumina and 454 sequencing methods and compared to the sequence of M. gallisepticum strain R (R_low_), a well-documented reference strain that is virulent in chickens (26, 29). In this study, the use of comparative genomics to identify strain-specific marker sequences that can distinguish between the M. gallisepticum ts-11 vaccine strain and natural field isolates is demonstrated.

## RESULTS

**FIG 1:**
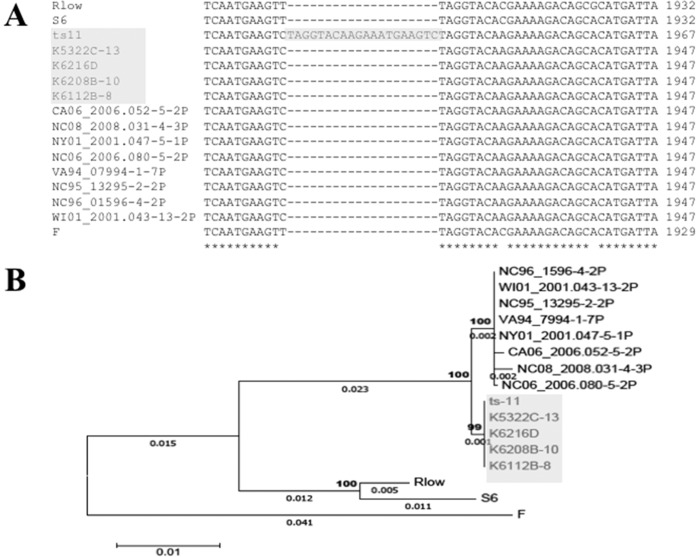
Sequence alignments and phylogenetic tree based on *gapA* adhesin. (A) A sequence alignment identified a 20-bp insertion (shaded in gray) in M. gallisepticum ts-11 within a conserved region of *gapA*. (B) Phylogenetic analysis confirmed that the gene sequences for *gapA* were identical in the M. gallisepticum ts-11 isolates (shaded in gray), except for this 20-bp insertion in the vaccine strain. The phylogenetic tree was constructed using the maximum-likelihood method based on the Tamura-Nei model. The tree is drawn to scale, with branch lengths measured in the number of substitutions per site (next to the branches). Bootstrap percentages after 1,000 replications and branch lengths of >0.001 are shown. The ts-11 isolates are shaded in gray.

**FIG 2:**
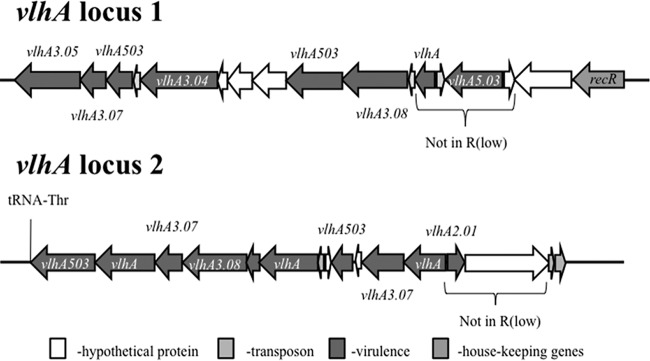
Identification of Mycoplasma gallisepticum ts-11 *vlhA* virulence loci. Forty of the virulence genes identified in M. gallisepticum were annotated as coding for the variable surface protein VlhA. These *vlhA* genes mapped to 6 loci within the ts-11 genome. Two of these loci are depicted, and locus 1 is of particular importance in that it houses the *vlhA* genes that were targeted for differentiating the ts-11 strain from natural field isolates.

**FIG 3:**
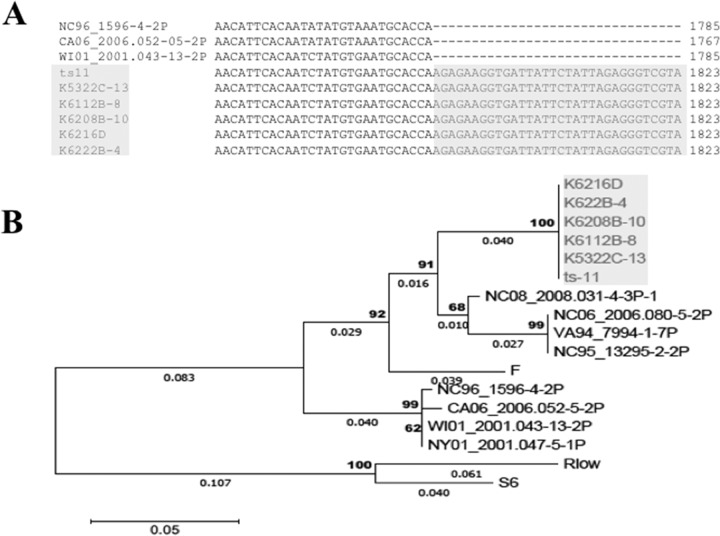
Identification of Mycoplasma gallisepticum ts-11-specific sequences in *vlhA*3.04b. (A) Sequences unique to the ts-11 isolates (shaded in gray) are contained in the 3′ region of this *vlhA*3.04b gene allele. (B) Phylogenetic analysis confirmed that M. gallisepticum ts-11 isolates (shaded in gray) grouped together due to conserved sequences. The phylogenetic tree was constructed using the maximum-likelihood method based on the Tamura-Nei model. The tree is drawn to scale, with branch lengths measured in the number of substitutions per site (next to the branches). Bootstrap percentages after 1,000 replications and branch lengths of >0.001 are shown.

**FIG 4:**
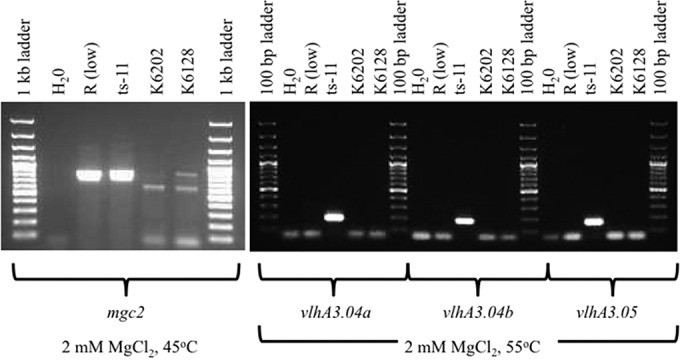
PCR to differentiate between ts-11 and field isolates. Five alleles were tested by PCR (*vlhA*3.04a, *vlhA*3.04b, *vlhA*3.05, *mg0359*, and *mg0377*). This gel depicts the results of three PCR tests (*vlhA*3.04a, *vlhA*3.04b, and *vlhA*3.05). K6202 and K6218 are field isolates (not ts-11 strain), and both were negative in the three PCR tests. All of the M. gallisepticum isolates were positive in the *mgc2* PCR.

Of the 803 annotated M. gallisepticum ts-11 genes, 70 were identified as having homology with putative virulence genes, including those involved in adherence. There was significant genetic diversity in two adhesins, encoded by *gapA* (Fig. 1) and *mcg2* (data not shown), among the analyzed M. gallisepticum genomes. No major genomic changes were identified among ts-11 avirulent and virulent isolates; there were numerous single-nucleotide polymorphisms (SNPs) that need to be further analyzed and confirmed. The gene sequences of *gapA* and *mcg2* were conserved in M. gallisepticum ts-11 isolates, except for the presence of a 20-bp insertion in *gapA* of the vaccine strain ts-11. Forty of the virulence genes identified in the M. gallisepticum ts-11 genome were annotated as coding for the variable surface protein VlhA, and these *vlhA* genes mapped to one of 6 loci within the ts-11 genome. Two of these loci are depicted in Fig. 2, and *vlhA* locus 1, which contains strain-specific sequences, is highlighted. Comparison of the *M. gallisepticum vlhA* genes among M. gallisepticum strains identified several *vlhA* genes containing sequences that were unique to ts-11 strains (Fig. 3). The *vlhA* alleles containing sequences unique to the ts-11 isolates included *vlhA*3.04a, *vlhA*3.04b, *vlhA*3.04d, *vlhA*3.05*c*, *vlhA*3.08a, and *vlhA*5.03b. In addition to these *vlhA* alleles, several other genes were identified that were unique to the ts-11 isolates, contained strain-specific sequences, or were limited in their distribution among M. gallisepticum genomes. Of these genes and gene sequences, five likely candidates were selected for strain-specific-typing PCR tests: *vlhA*3.04a, *vlhA*3.04b, *vlhA*3.05, *mg0359*, and *mg0377*. The PCR protocols were optimized, and no evidence of nonspecific amplification or additional bands was identified when the gels were analyzed (Fig. 4). A summary of the PCR analysis of the M. gallisepticum isolates is presented in Table 1. All M. gallisepticum ts-11 isolates were positive for all five alleles tested by PCR (*vlhA*3.04a, *vlhA*3.04b, *vlhA*3.05, *mg0359*, and *mg0377*). There was a significant difference (*P* < 0.005) in the distribution of these alleles in M. gallisepticum isolates (ts-11 versus field isolates). However, no single allele was unique to the ts-11 strain compared to the field isolates screened in this study. The distribution of the alleles in field isolates ranged between 5% and 36%. The *vlhA*3.05 PCR had the highest discriminatory power, followed by the *mg0359* PCR (5% and 7% of non-ts-11 isolates were positive, respectively). A combination of primers (*vlhA*3.04a, *vlhA*3.05, and *mg0359*) was able to differentiate ts-11 isolates from M. gallisepticum field isolates (100% versus 0%, respectively).

**TABLE 1:** Distribution of ts-11 specific genes and alleles in M. gallisepticum isolates

PCR Target(s)	No. of strains positive/total no. of strains (%)		
	ts-11 isolates	Others	Prob >X^2^
*vlhA 3.04a*	11/11 (100%)	10/42 (24%)	<0.0001
*vlhA 3.04b*	11/11 (100%)	15/42 (36%)	0.0001
*vlhA 3.05*	11/11 (100%)	2/42 (5%)	<0.0001
*mg0377*	11/11 (100%)	14/42 (33%)	<0.0001
*mg0359*	11/11 (100%)	3/42 (7%)	<0.0001
*mgc2*	11/11 (100%)	42/42 (100%)	
*vlhA 3.04a, vlhA 3.05*, *mg0359*	11/11 (100%)	0/42 (0%)	

## DISCUSSION

The inability to distinguish M. gallisepticum field isolates from vaccine strains has made it difficult to understand the epidemiology of avian mycoplasmosis, especially when there is an outbreak involving vaccinated flocks. Methods that employ DNA fingerprinting require the isolation of pure cultures (15) and can only differentiate M. gallisepticum ts-11 from a few other strains (14, 33). Other methods, such as gene-targeted sequencing, require the use of PCR followed by sequencing and sequence alignments (23), a time- and resource-intensive process.

Although no major genomic differences that could differentiate ts-11 virulent from ts-11 avirulent isolates were identified, this study revealed that the M. gallisepticum ts-11 vaccine and ts-11 isolates, obtained from the field and from ts-11 animal passage experiments, exhibited genomic differences compared to the M. gallisepticum R_low_ genome in the form of insertions/deletions of sequences. In some cases, entire gene islets (consisting of up to four genes) were seen in ts-11 isolates that are absent in R_low_. *vlhA* locus 1 contained a large number of *vlhA* genes that were shown to contain genotypic differences among the strains. This was essential in allowing for the identification of sequences that distinguish the M. gallisepticum ts-11 strain from field isolates. Phylogenetic analysis, carried out using each of these strain-typing candidate genes, showed that all the ts-11 isolates clustered, indicating there was sufficient sequence variability to formulate a discriminatory PCR-based test.

Multiple sequence alignments allowed for the rapid identification of genomic differences between the M. gallisepticum ts-11 isolates and the virulent R_low_ strain. One such difference was the identification of a 20-bp insertion in *gapA* of the vaccine strain ts-11. This insertion has been predicted to disrupt the protein and affect the adherence capability of M. gallisepticum, and consequently, this insertion in GapA affects the colonization of chickens by the vaccine strain (34). The insertional mutation was lost in the M. gallisepticum ts-11 field isolates, suggesting that the vaccine can revert to wild type. Studies have reported reversion of GapA^−^ to GapA^+^ when ts-11 is passaged in chickens, resulting in an increased ability of the bacteria to colonize. However, GapA^+^ ts-11 isolates are not pathogenic (18, 34), indicating that additional changes are necessary for full reversion to the virulent phenotype.

Most of the other genes identified as candidates for ts-11-specific detection were *vlhA* genes. These encode variable surface lipoproteins and contain significant interstrain sequence variation (16). This genetic diversity is not surprising, as mycoplasmas have been known to have high mutation rates (24). This variability is introduced by various mechanisms, such as insertions, deletions, or rearrangements. *vlhA* locus 1 is the main locus in which many *vlhA* genes exhibited significant sequence diversity. The *vlhA*3.04a, -3.04b, and -3.05 genes from this locus were chosen because they contained sequences that were unique to the M. gallisepticum ts-11 isolates and highly conserved within this group. The *vlhA*3.05 gene possessed significant differentiating power, identifying all ts-11 isolates (all of these were positive), while only 5% of the field isolates tested positive. To achieve optimal discriminatory power, a combination of *vlhA*3.05, *mg0359*, and *vlhA*3.04a PCR tests was effective in differentiating M. gallisepticum ts-11 isolates from natural field isolates.

This study describes a useful molecular assay that can differentiate at a high level between the M. gallisepticum ts-11 strain and field isolates. This will supplement current tools available for diagnostics and is necessary for determining strain interactions, evaluating vaccination programs, and controlling the spread of M. gallisepticum within poultry complexes. This work will allow differentiation between an attenuated vaccine strain and field strains, thereby providing the tools necessary to rapidly recognize field challenges.

## MATERIALS AND METHODS

### M. gallisepticum isolates.

The ts-11 vaccine and ts-11 reisolates used in this study are listed in Table 2. The vaccine was obtained from the manufacturer (Mycoplasma gallisepticum vaccine, serial no. MA649; Merial Select, Gainesville, GA, USA). The field isolates were recovered from clinical submissions to the Poultry Diagnostic and Research Center (PDRC), University of Georgia, Athens, GA USA, during a period from October 2007 to February 2010. The isolates were genotyped as ts-11 strains based on multiple genetic analyses, including targeted sequencing and random amplified polymorphic DNA (RAPD) analysis (9). The clinical picture was obtained from history at submission and/or interviews with field veterinarians. Bird trials confirmed the pathogenicity of two of the isolates (K6212D and K6222B) (9, 32). One reisolate (K5322C-13) was obtained from specific-pathogen-free layer-type chickens at 2 weeks postvaccination with ts-11. Three additional M. gallisepticum ts-11 isolates from the field were tested by the PCR protocols developed, as well as 42 additional strains, including 6 reference (PG-31, R_low_, A5969, and S-6) and vaccine (F and 6/85) strains and 15 field isolates (1998 to 2011) from the United States. International isolates from Jordan (2 isolates), India (2 isolates), Israel (2 isolates), Slovenia (6 isolates), Brazil (1 isolates), Germany (3 isolates), Mexico (1 isolates), and the Netherlands (4 isolates) were also tested. These M. gallisepticum isolates were obtained from the PDRC repository.

**TABLE 2:** Description of M. gallisepticum ts-11 isolates used in this study

Isolate	Description^*a*^	Virulence	GenBank Accession^*b*^	Reference
ts-11 (K2966)	Vaccine (attenuated strain)	Avirulent	MAFU00000000	(7)
K5322C-13	Re-isolate from SPF layers; No CS; V	Avirulent	MAFV00000000	This study
K6112B-8	BB; No CS; V	Avirulent	MAFW00000000	This study
K6208B-10	BB; No CS; V	Avirulent	MADW00000000	This study
K6216D	B; CS; NV	Virulent	MATM00000000	(9, 32)
K6222B	BB; CS; V	Virulent	MATN00000000	(9, 32)
K6356-12	B; CS; NV	Virulent	MAGQ00000000	This study
K6372-23	B; CS; NV	Virulent	MAGR00000000	This study

a V–Vaccinated; NV–Not Vaccinated; B–Broiler chickens; BB–Broiler breeder chickens; CS–Clinical signs (of respiratory disease). Virulence was determined by pathogenicity trials and clinical picture (9, 32). All broiler chickens were progeny of ts-11-vaccinated breeders.

b Whole Genome Sequence, NCBI GenBank Bioproject # PRJNA325637.

### DNA extraction.

M. gallisepticum isolates were grown as previously described (9). DNA was extracted from M. gallisepticum isolates as follows: after growth in Frey's modified broth, cells were centrifuged at 13,000 × *g* for 3 min, the supernatant discarded, and the cell pellets reconstituted in 200 μl of phosphate-buffered saline (pH 7). Genomic DNA was extracted using the Qiagen DNeasy blood and tissue kit (Qiagen, Valencia, CA) following the manufacturer's recommendations.

### Whole-genome sequencing, alignment, and annotation.

The sequenced isolates are listed in Table 2. Whole-genome libraries were produced using Illumina, following the manufacturer's protocols to produce paired-end 151-bp reads. Raw sequence data were screened for adapter contamination and quality using FastQC (http://www.bioinformatics.babraham.ac.uk/projects/fastqc/). The sequence libraries were quality trimmed to remove low-quality bases using FastaQ/A Trimmer in the FASTX-Toolkit (http://hannonlab.cshl.edu/fastx_toolkit/index.html). The best k-mer length for *de novo* assembly was determined using KmerGenie (35). *De novo* assembly of paired-end Illumina reads was done using Velvet (36). Reads obtained by the Roche 454 sequencing protocol were assembled using GS *De Novo* Assembler (http://www.454.com/products/analysis-software/). Contigs from each isolate library were ordered against the complete genome of M. gallisepticum R_low_ (GenBank accession number NC_004829.2), which served as the reference genome for this study (26), using the Mauve Genome Alignment Tool (37). Contigs were renamed using Perl scripting to reflect the order derived from the reference genome. Each library was then annotated using the RAST annotation server.

### Bioinformatic analysis.

Sequence-based analysis of the annotated genomes was accomplished using RAST Seed Viewer (http://theseed.org). Each isolate's genome was compared to the M. gallisepticum R_low_ reference genome in order to identify genes that were absent in R_low_, present in M. gallisepticum ts-11 vaccine and ts-11 reisolate genomes, or had <80% similarity to the corresponding gene in the reference genome. Jalview (38) was used to create multiple sequence alignments of each candidate ts-11-specific gene, comparing the genomes of M. gallisepticum ts-11 vaccine strain and ts-11 isolates to published M. gallisepticum genomes (strain S6, GenBank accession number NC_023030.1; strain F, GenBank accession number NC_017503.1; and house finch isolates, GenBank accession numbers NC_018412.1, NC_018409.1, NC_018406.1, NC_018407.1, NC_018408.1, NC_018410.1, NC_018411.1, and NC_018413.1). Nucleotide sequences found to be conserved in the ts-11 vaccine strain and ts-11 reisolates were then searched against the NCBI database using BLAST to test their specificity *in silico*. Phylogenetic trees were constructed using the neighbor-joining method (Jalview) and the maximum-likelihood method based on the Tamura-Nei model in MEGA6 (39) in order to assess the evolutionary similarities among the M. gallisepticum ts-11 vaccine strain, the ts-11 reisolates, and other M. gallisepticum isolates. Nucleotide and amino acid sequences for M. gallisepticum strain-specific genes/alleles, as well as *gapA* and *mgc2*, were submitted to National Center for Biotechnology Information (NCBI) GenBank (http://www.ncbi.nlm.nih.gov/GenBank/) under GenBank accession numbers KU577580 to KU577613. In addition, the M. gallisepticum whole-genome sequences were deposited with NCBI GenBank (Bioproject accession number PRJNA325637).

### PCR.

PCR primers were designed with the web-based software Primer3Plus (http://www.bioinformatics.nl/cgi-bin/primer3plus/primer3plus.cgi), using strain-specific sequences identified in *vlhA*3.04a, *vlhA*3.04b, *vlhA*3.05, *mg0359*, and *mg0377*. PCR was performed using a RapidCycler thermocycler (Idaho Technologies; Idaho Falls, ID) (40) in 10-μl-capacity capillary tubes; the primers are listed in Table 3. The PCR mixture consisted of 0.2 mM deoxynucleoside triphosphates (dNTPs), 2.0 mM MgCl_2_ (with *vlhA*3.04a, *vlhA*3.04b, *vlhA*3.05, and *mgc2* primers) or 3.0 mM MgCl_2_ (with *mg0359* and *mg0377* primers), 0.5 μM each primer, 0.5 unit of *Taq* DNA polymerase (Invitrogen, Waltham, MA), and 1 μl DNA template in a 10-μl volume. The program parameters for the thermocycler were 94°C for 0 s, 55°C for 0 s, and 72°C for 15 s with a slope of 2.0 for 30 cycles. For *mgc2*, the program parameters were 94°C for 1 min, then 94°C for 10 s, 45°C for 10 s, and 72°C for extension for 35 s with a slope of 2.0 for 30 cycles.

**TABLE 3:** PCR primers

Target	Primer Sequence	Expected size (bp)	MgCl_2_ (mM)	Annealing Temperature (°C)	Reference
*vlhA 3.04a*	F: tactgaaaacgctgatggac	187	2	55	This study
	R: gccactagttcctgctgcat				
*vlhA 3.04b*	F: gggtcgtatcttacaaatgcac	180	2	55	This study
	R: tccatcagcgtttgcagtag				
*vlhA 3.05*	F: catccgataatgtagggcttg	158	2	55	This study
	R: tgcagagctagattgatttcca				
*mg0377*	F: ctgaaaaatccagggggtct	176	3	55	This study
	R: tgctgattgagtggatttcg				
*mg0359*	F: gggagacagagcaagaaatatca	229	3	55	This study
	R: agggaacaatttatctcaatctgaa				
*mgc2*	F: gctttgtgttctcgggtgcta	824	2	45	(40)
	R: cggtggaaaaccagctcttg				

Amplicons were separated by gel electrophoresis in a 1.5% agarose gel (15 by 10 cm) with 0.5 μg ethidium bromide/ml and 1× TAE (40 mM Tris, 20 mM acetate, 1 mM EDTA, pH 8) buffer at a constant voltage of 80 V for 60 min. A 100-bp ladder (DNA molecular weight marker; Roche, Indianapolis, IN) was used as the molecular weight (MW) standard for determining the MW of the PCR amplicons. The agarose gels were exposed to ultraviolet light and photographed using a digital photo documentation system (Molecular Imager Gel Doc XR system; Bio-Rad Laboratories, San Diego, CA). The discriminatory power of each PCR to distinguish the vaccine strain (ts-11) from field isolates of M. gallisepticum was determined by Pearson's chi-square test in JMP Statistics Made Visual (SAS Institute, Inc., Cary, NC) (*P* < 0.005).
